# Midterm Efficacy of SubcutAneous Implantable CardioVErter‐Defibrillator in ≤ 18 Year‐Old CHILDREN (SAVE CHILDREN‐II Registry)

**DOI:** 10.1002/joa3.70420

**Published:** 2026-07-13

**Authors:** Hitoshi Mori, Taisuke Nabeshima, Kota Nagaoka, Hideo Fukunaga, Hidemori Hayashi, Osamu Inaba, Yuhei Isonaga, So Asano, Junichi Nitta, Shigeo Watanabe, Sou Otsuki, Hiroshi Suzuki, Hidehira Fukaya, Jun Kishihara, Toshiyuki Chisaka, Takashi Higaki, Ayako Okada, Hideki Kobayashi, Takayuki Sekihara, Takafumi Oka, Seiichi Sato, Keisuke Yoshino, Hidekazu Kondo, Naohiko Takahashi, Ken Kato, Masaru Miura, Jun Maeda, Shuntaro Tamura, Kentaro Ikeda, Hiroko Goto, Takashi Murakami, Tsutomu Wada, Motoki Takamuro, Tsugutoshi Suzuki, Yuji Ishida, Shingo Sasaki, Takuro Nishimura, Masateru Takigawa, Tomomi Matsuura, Tomohiko Imamura, Satoshi Shizuta, Masato Fukunaga, Maiko Kuroda, Rintaro Hojo, Seiji Fukamizu, Hirohiko Suzuki, Yukihiko Yoshida, Itsuro Morishima, Kenji Kuraishi, Eiki Nishihara, Yoshihisa Naruse, Tomoyuki Sato, Yoshiyasu Aizawa, Kimitaka Nishizaki, Kentaro Nakamura, Norihito Nuruki, Masao Yoshinaga, Yukitoshi Ikeya, Yasuo Okumura, Shota Muraji, Yusuke Kondo, Makoto Nakano, Machiko Kito, Yasuya Inden, Satoshi Higuchi, Takahiko Kinjo, Kazuhisa Matsumoto, Masataka Narita, Wataru Sasaki, Tsukasa Naganuma, Daisuke Kawano, Naomichi Tanaka, Kei Matsumoto, Takuro Kojima, Yoshifumi Ikeda, Ritsushi Kato, Naokata Sumitomo

**Affiliations:** ^1^ Department of Pediatric Cardiology Saitama Medical University International Medical Center Saitama Japan; ^2^ Department of Cardiology Saitama Medical University International Medical Center Saitama Japan; ^3^ Division of Cardiology Showa University School of Medicine Tokyo Japan; ^4^ Department of Pediatrics and Adolescent Medicine Juntendo University Graduate School of Medicine Tokyo Japan; ^5^ Department of Cardiovascular Biology and Medicine Juntendo University Graduate School of Medicine Tokyo Japan; ^6^ Department of Cardiology Japanese Red Cross Saitama Hospital Saitama Japan; ^7^ Department of Cardiology Sakakibara Heart Institute Tokyo Japan; ^8^ Department of Pediatric Cardiology Yokohama City University Hospital Yokohama Kanagawa Japan; ^9^ Department of Cardiovascular Biology and Medicine Niigata University Graduate School of Medical and Dental Sciences Niigata Japan; ^10^ Uonuma Institute of Community Medicine Niigata University Medical and Dental Hospital Niigata Japan; ^11^ Department of Cardiovascular Medicine Kitasato University School of Medicine Sagamihara Kanagawa Japan; ^12^ Department of Regional Child Health Care Ehime University Graduate School of Medicine Matsuyama Ehime Japan; ^13^ Department of Pediatric and Adolescent Therapeutic and Developmental Education Ehime University Graduate School of Medicine Matsuyama Ehime Japan; ^14^ Department of Cardiovascular Medicine Shinshu University School of Medicine Nagano Japan; ^15^ Department of Cardiovascular Medicine The University of Osaka Graduate School of Medicine Osaka Japan; ^16^ Department of Pediatric Cardiology Okinawa Prefectural Nanbu Medical Center and Childrens Medical Center Okinawa Japan; ^17^ Department of Cardiology and Clinical Examination Oita University Faculty of Medicine Yufu City Oita Japan; ^18^ Department of Cardiology Tokyo Metropolitan Tama Medical Center Tokyo Japan; ^19^ Department of Cardiology Tokyo Metropolitan Children's Medical Center Tokyo Japan; ^20^ Department of Cardiovascular Medicine Gunma University Graduate School of Medicine Maebashi City Gunma Japan; ^21^ Department of Cardiology Gunma Children's Medical Center Shibukawa City Gunma Japan; ^22^ Department of Pediatric Cardiology, Department of Cardiology Nagoya Tokushukai General Hospital Kasugai‐shi Aichi Japan; ^23^ Department of Pediatric Cardiology Gifu Prefectural General Medical Center Gifu Japan; ^24^ Department of Child Health, Institute of Medicine University of Tsukuba Ibaraki Japan; ^25^ Department of Pediatrics Sapporo Medical University Sapporo Hokkaido Japan; ^26^ Department of Pediatric Cardiology Hokkaido Medical Center for Child Health and Rehabilitation Sapporo Hokkaido Japan; ^27^ Department of Pediatric Electrophysiology Osaka City General Hospital Osaka Japan; ^28^ Department of Cardiology and Nephrology Hirosaki University Graduate School of Medicine Aomori Japan; ^29^ Department of Cardiovascular Medicine Institute of Science Tokyo Tokyo Japan; ^30^ Department of Cardiovascular Medicine Tokushima University Tokushima Japan; ^31^ Department of Preventive Services, School of Public Health Kyoto University Kyoto Japan; ^32^ Department of Cardiovascular Medicine, Graduate School of Medicine Kyoto University Kyoto Japan; ^33^ Department of Cardiology Kokura Memorial Hospital Fukuoka Japan; ^34^ Department of Cardiology Tokyo Metropolitan Hiroo Hospital Tokyo Japan; ^35^ Department of Cardiology Japanese Red Cross Aichi Medical Center Nagoya Daini Hospital Nagoya Aichi Japan; ^36^ Department of Cardiology Ogaki Municipal Hospital Gifu Japan; ^37^ Department of Pediatric Cardiology and Neonatology Ogaki Municipal Hospital Gifu Japan; ^38^ Division of Cardiology, Internal Medicine III Hamamatsu University School of Medicine Shizuoka Japan; ^39^ Department of Pediatrics Jichi Medical University Tochigi Japan; ^40^ Department of Cardiovascular Medicine Nippon Medical School Hospital Tokyo Japan; ^41^ Department of Cardiology Teine Keijinkai Hospital Sapporo Hokkaido Japan; ^42^ Department of Cardiology Urasoe General Hospital Okinawa Japan; ^43^ Department of Arrhythmia Treatment NHO Kagoshima Medical Center Kagoshima Japan; ^44^ Department of Pediatrics NHO Kagoshima Medical Center Kagoshima Japan; ^45^ Division of Cardiology Nihon University Itabashi Hospital Tokyo Japan; ^46^ Department of Cardiology Fukuoka Children's Hospital Fukuoka Japan; ^47^ Department of Cardiology Chiba University Chiba Japan; ^48^ Department of Cardiovascular Medicine Tohoku University Graduate School of Medicine Sendai Miyagi Japan; ^49^ Department of Pediatric Cardiology Aichi Children's Health and Medical Center Obu Aichi Japan; ^50^ Department of Cardiology Nagoya University Graduate School of Medicine Showa Aichi Japan; ^51^ Clinical Research Division for Heart Rhythm Management, Department of Cardiology Tokyo Women's Medical University Tokyo Japan; ^52^ Department of Pediatric Cardiology and Adult Congenital Cardiology Tokyo Women's Medical University Tokyo Japan

**Keywords:** children, implantable cardioverter‐defibrillator, subcutaneous implantable cardioverter defibrillator, sudden cardiac death

## Abstract

**Background:**

The subcutaneous implantable cardioverter‐defibrillator (S‐ICD) avoids transvenous leads and is a promising option for sudden cardiac death (SCD) prevention in pediatric patients. However, mid‐term outcomes and post‐shock management strategies remain insufficiently characterized.

**Methods:**

This multicenter, retrospective observational study included pediatric patients (≤ 18 years) who underwent S‐ICD implantation between February 2016 and July 2021. Clinical characteristics, pre‐implant screening, procedural details, device‐related events, and follow‐up data were analyzed. The incidence and management of appropriate and inappropriate therapies and subsequent recurrence were evaluated.

**Results:**

Ninety‐six patients (median age 14.5 years) were enrolled and followed for a median of 70 months (29–75.0 months). Sensing vector suitability remained stable despite somatic growth. Appropriate shocks occurred in 32 patients (33.7%), while inappropriate shocks occurred in 27 (28.4%). After appropriate therapy, intensified pharmacological treatment and catheter ablation prevented recurrent device therapy in 45.5% and 50.0% of cases, respectively, although device shock occurred in 50.9% despite intervention. Following inappropriate therapy, device reprogramming and lifestyle guidance prevented recurrence in 71.4% of patients (15/21). Device‐related infection was rare (2 cases), and no lead fractures were observed.

**Conclusions:**

S‐ICD therapy demonstrated favorable mid‐term safety and efficacy in pediatric patients, with durable sensing performance and a low incidence of device‐related infection. Although inappropriate shocks were not uncommon, appropriate post‐shock management effectively reduced recurrence, supporting S‐ICD as a viable option for selected pediatric patients without pacing requirements.

## Graphical abstract

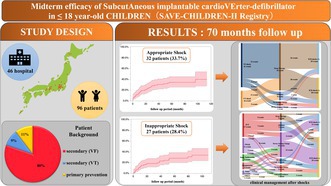

**Abbreviations**
CPVT: catecholaminergic polymorphic ventricular tachycardia
DFT: defibrillation threshold
ECG: electrocardiogram
EV‐ICD: extravascular implantable cardioverter‐defibrillator
ICD: implantable cardioverter‐defibrillator
IQR: interquartile range
LQTS: long QT syndrome
SCD: sudden cardiac death
S‐ICD: subcutaneous implantable cardioverter‐defibrillator
SVT: supraventricular tachycardia
TV‐ICD: transvenous implantable cardioverter‐defibrillator
TWOS: T‐wave oversensing
VF: ventricular fibrillation
VT: ventricular tachycardia

## 1 Introduction

The implantable cardioverter‐defibrillator (ICD), which includes transvenous (TV‐ICD), subcutaneous (S‐ICD), and extravascular (EV‐ICD) systems, is an established therapy for preventing sudden cardiac death (SCD) caused by lethal arrhythmias such as ventricular tachycardia (VT) and ventricular fibrillation (VF) [1, 2, 3, 4, 5, 6]. However, most clinical evidence supporting SCD prevention is derived from studies in adults, with limited data available for pediatric patients [7, 8, 9]. In pediatric populations, long‐term device management poses unique challenges, including lead stress due to growth and an increased risk of infection over time [8, 10, 11, 12]. Furthermore, the underlying cardiac diseases often differ from those in adults, and many pediatric patients do not require bradycardia pacing therapy [1].

The S‐ICD has recently emerged as a promising option for pediatric SCD prevention, offering a lower risk of long‐term complications such as device‐related endocarditis, tricuspid regurgitation, and superior lead durability [13, 14, 15, 16]. We previously demonstrated the procedural safety and acute efficacy of the S‐ICD in pediatric patients [17]; however, long‐term outcomes remain insufficiently characterized. The median follow‐up of our study was only 27 months, and data on sustained safety, efficacy, and post‐shock management were limited. Appropriate shocks occurred in 26.2% of patients and inappropriate shocks in 21.3%, yet subsequent treatment strategies and outcomes were unclear.

Therefore, this study aimed to evaluate the mid‐term safety, efficacy, and clinical outcomes following appropriate and inappropriate S‐ICD shocks in the pediatric population.

## 2 Methods

This multicenter, observational, and retrospective study investigated patients who underwent S‐ICD implantation from February 2016 to July 2021, corresponding to that of the SAVE‐CHILDREN study [17]. All included patients, except those who expressed their refusal to participate through the opt‐out process, were 18 years of age or younger at the time of implantation.

This study protocol was approved by the institutional review board of Saitama Medical University International Medical Center (approval number: 2024‐129) through a centralized review process.

### 2.1 Data Collection

This study focused on safety, efficacy, and long‐term clinical outcomes following appropriate and inappropriate shocks delivered by S‐ICD, over a longer follow‐up period than that reported in previous studies. The following data were collected:

1. Patients' background characteristics
2. Pre‐screening test for S‐ICD

Eligibility for S‐ICD therapy was evaluated before implantation through a standardized pre‐screening process. This assessment was performed using an ECG‐based screening tool or a proprietary programmer provided by Boston Scientific (Marlborough, MA, USA), and the corresponding pre‐screening data were collected. We evaluated the pass rates of the primary, secondary, and alternate sensing vectors, as well as the number of vectors that successfully passed the screening process.

3 Procedure details

Device implantation was performed under general anesthesia or deep sedation. The implantation side was determined on the basis of pre‐screening results. The choice between a three‐incision and a two‐incision technique was determined by the operator's discretion, with consideration given to patient body size and general condition [18]. When the subcutaneous lead was disproportionately large relative to the patient's body size, the electrode insertion tool (tunneler) was manually bent to ensure optimal positioning of the distal lead tip [17, 19, 20]. Data on post‐implantation sensing vector selection, shock polarity, and therapy programming were collected. Complications were classified as acute if they occurred within 30 days after implantation and as chronic if they developed thereafter.

4 Follow‐up data

Patients underwent outpatient follow‐up after device implantation. Data on changes in age and body size until the final follow‐up and on sensing vector compatibility evaluated using the device programmer were collected and analyzed.

The incidence of first appropriate and inappropriate device therapies during follow‐up was assessed for each patient. After each appropriate or inappropriate therapy, data regarding the underlying arrhythmia and subsequent clinical management were collected. The occurrence of recurrent device therapy after subsequent clinical management was also analyzed for each event.

5 Device Related Events

Device‐related events were categorized into acute‐phase events, defined as those occurring within 30 days after implantation, and chronic‐phase events, defined as those occurring more than 30 days after implantation, and data were collected accordingly.

Because a recall related to battery malfunction of the S‐ICD occurred in 2020, cases involving implantation of recalled devices were identified and analyzed as recalled‐device cases. In addition, regardless of recall status, cases requiring early battery replacement were classified as early device depletion and were included as such in the data collection.

### 2.2 Statistical Analyses

The statistical analyses were performed using JMP Pro, version 18 software (SAS Institute) and Python (Python 3.11.0) software. The Shapiro–Wilk test was used to assess data distribution, and continuous variables are presented as medians with interquartile ranges (IQRs). Continuous variables were compared using the two‐tailed Mann–Whitney U test, while categorical variables were compared using the chi‐square test. Time‐to‐event analyses for device therapies were performed using the Kaplan–Meier method. A *p* value < 0.05 indicated statistical significance.

## 3 Results

### 3.1 Baseline Patient Characteristics

A total of 96 patients were enrolled from 46 centers. Table 1 shows the patients details. The patients ranged in age from 3 to 18 (median 14.5 years old [IQR 12.0–17.0 years]). The body height and body weight at the time of ICD implantation ranged from 86 cm to 183 cm (median 160.3 cm [IQR 149.6 cm to 170.0 cm]) and from 11 kg to 89 kg (median 50.0 kg [IQR 43.0 kg to 57.7 kg]), respectively. The median left ventricular ejection fraction was 64.0% (58.0%–71.0%). An electrophysiological study was performed prior to device implantation in 20 patients (20.8%), among whom VT/VF was inducible in 8 patients (40.0%). Exercise stress testing was conducted in 55 patients (57.3%), with a maximum heart rate ranging from 104 to 237 beats per minute (bpm) (median, 164 bpm [129.5–181 bpm]). Overall, 62 patients (64.6%) were receiving β‐blocker therapy, and 36 (36.5%) were treated with antiarrhythmic medications. In contrast, 23 patients (24.0%) were not receiving pharmacological therapy.

**TABLE 1:** Patient characteristics.

Clinical parameters	*n* = 96
Age at implantation, years (range)	14.5 (12.0–17.0)
Gender, male, *n* (%)	68 (70.8)
Body height, cm (range)	160.3 (149.6–170.0)
Body weight, kg (range)	50.0 (43.0–57.7)
Median follow up, months (range)	70.0 (29.0–75.0)
LVEF, % (range)	64.0 (58.0–71.0)

Medical therapy	Patients	Drug dose
ß‐blocker, *n* (%)	62 (64.6)	
Bisoprolol, *n* (%), mg	33 (53.2)	2.5 (1.25–4.0)
Carvedilol, *n* (%), mg	12 (19.4)	10 (6.25–18.8)
Nadolol, *n* (%), mg	9 (14.5)	30 (22.5–30)
Propranolol, *n* (%), mg	6 (9.7)	40 (30–100)
Atenolol, *n* (%), mg	1 (1.6)	50
Metoprolol, *n* (%), mg	1 (1.6)	80
Antiarrhythmic medications, *n* (%)	36 (36.5)^a^	
Amiodarone, *n* (%), mg	11 (30.6)	160 (100–200)
Mexiletine, *n* (%), mg	9 (25.0)	300 (150–300)
Flecainide, *n* (%), mg	6 (16.6)	85 (57.5–162.5)
Sotalol, *n* (%), mg	4 (11.1)	160 (100–160)
Cibenzoline, *n* (%), mg	3 (8.3)	150 (100–160)
Verapamil, *n* (%), mg	2 (5.6)	80–240
Quinidine, *n* (%), mg	1	450

*Note:* The continuous variables are shown as median (IQR) and categorical variables as the number (%).

Abbreviation: LVEF, left ventricular ejection fraction.

^a^ One patient took mexiletine and verapamil.

**FIGURE 1:**
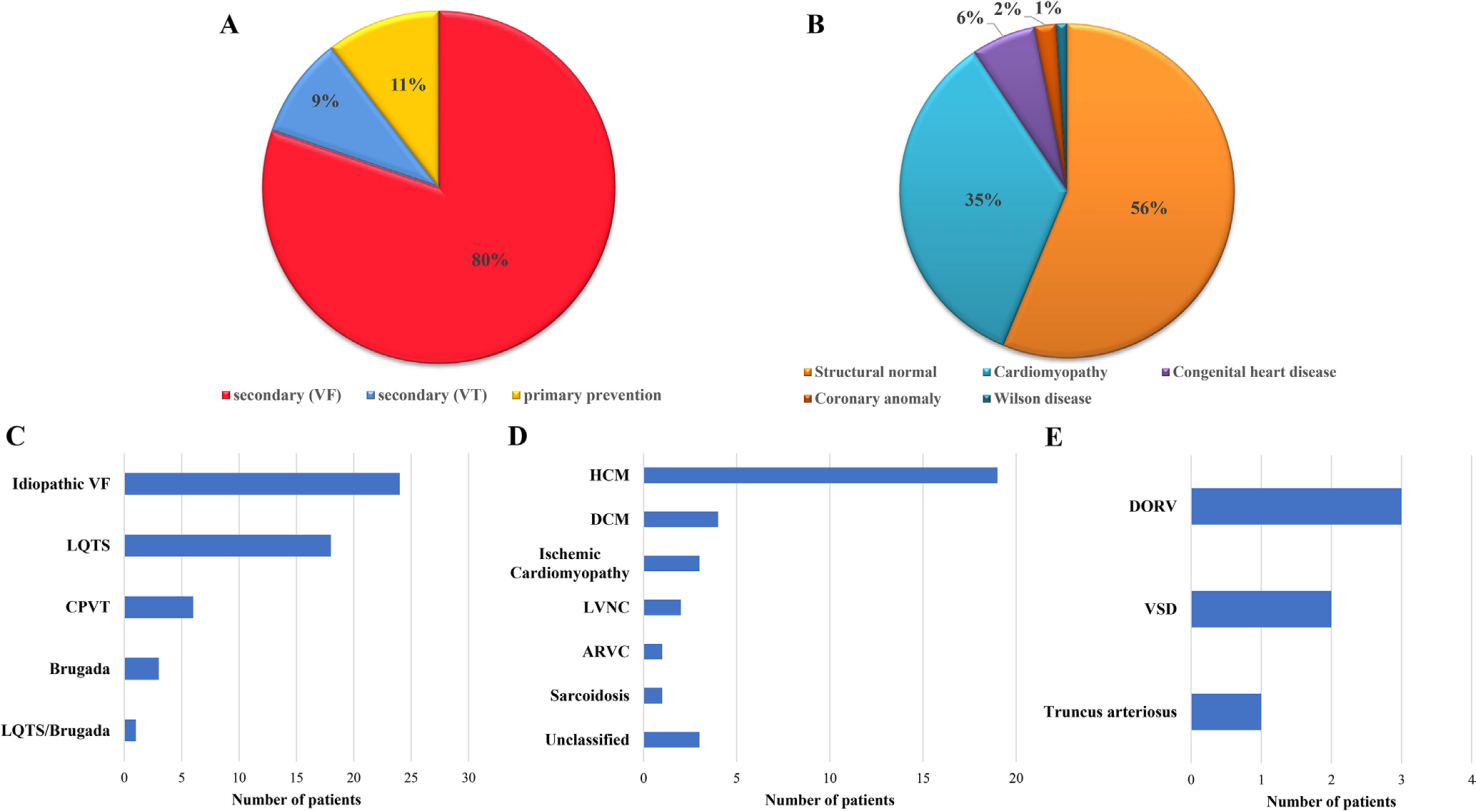
The indications for ICD implantation and underlying cardiac diagnoses are shown in Figure 1. Secondary prevention for VF accounted for the majority of cases (80%), whereas primary prevention accounted for 11% (A). Regarding underlying cardiac diseases, primary electrical disorders without structural heart disease were the most prevalent category (B). Among these, idiopathic VF was the most common diagnosis (C). Among cardiomyopathies, HCM was the most frequently observed subtype (D). With respect to congenital heart disease, DORV was the most common underlying condition (E). ARVC, arrhythmogenic right ventricular cardiomyopathy; CPVT, catecholaminergic polymorphic ventricular tachycardia; DCM, dilated cardiomyopathy; DORV, double outlet right ventricle; HCM, hypertrophic cardiomyopathy; LQTS, long‐QT syndrome; LVNC, left ventricular noncompaction; VF, ventricular fibrillation; VSD, ventricular septal defect.

Figure 1 shows the baseline heart diseases. Seventy‐seven (80.2%) underwent S‐ICD implantation for secondary prevention of VF, and 9 (9.0%) for secondary prevention of VT. Ten patients (11.0%) underwent S‐ICD implantation for primary prevention of SCD. Primary prevention ICD implantation was performed only in patients aged 13 years or older, whereas all implantations in patients aged 12 years or younger were for secondary prevention (Figure S1). A total of 33 patients (34.4%) had cardiomyopathy, with hypertrophic cardiomyopathy being the most prevalent subtype. Fifty‐four patients (56.3%) had structurally normal hearts, with primary electrical disorders—including long QT syndrome (LQTS), idiopathic ventricular fibrillation, catecholaminergic polymorphic ventricular tachycardia (CPVT), and Brugada syndrome—identified as the underlying conditions. Among these, LQTS was the most common primary electrical disorder.

### 3.2 Pre‐Screening Test

Table 2 presents the pre‐screening results. Screening was predominantly conducted on the left side. The secondary vector showed the highest pass rate regardless of body position or screening side. Most patients passed screening with all vectors, while failure of all vectors was rare.

**TABLE 2:** Pre‐screening results.

Supine position	Left side	Right side	*p*
	*n* = 75	*n* = 56	
Primary, pass, *n* (%)	54/75 (72.0)	41/56 (73.2)	0.88
Secondary, pass, *n* (%)	64/75 (85.3)	45/56 (80.4)	0.45
Alternate, pass, *n* (%)	53/75 (70.7)	43/56 (76.8)	0.43
Passing vectors, 3, *n* (%)	37/75 (49.3)	27/56 (48.2)	0.90
Passing vectors, 2, *n* (%)	24/75 (32.0)	20/56 (35.7)	0.66
Passing vectors, 1, *n* (%)	12/75 (16.0)	8/56 (14.3)	0.79
Passing vectors, 0, *n* (%)	2/75 (2.7)	1/56 (1.8)	0.74

Sitting position	Left side	Right side	*p*
	*n* = 74	*n* = 53	
Primary, pass, *n* (%)	55/74 (74.3)	40/53 (75.5)	0.88
Secondary, pass, *n* (%)	61/74 (82.4)	42/53 (79.2)	0.65
Alternate, pass, *n* (%)	51/74 (68.9)	39/53 (73.6)	0.59
Passing vectors, 3, *n* (%)	34/74 (45.9)	25/53 (47.2)	0.89
Passing vectors, 2, *n* (%)	26/74 (35.1)	20/53 (37.7)	0.76
Passing vectors, 1, *n* (%)	13/74 (17.6)	6/53 (11.3)	0.33
Passing vectors, 0, *n* (%)	1/74 (1.4)	2/53 (3.8)	0.38

### 3.3 Procedure Detail

Table 3 shows the procedural data. The median procedure time was 95 min (78–129 min). Most patients underwent implantation under general anesthesia, although deep sedation was used in approximately one‐fifth of cases. Left‐sided implantation and the two‐incision technique were most frequently adopted. Tunneler bending was required in 9.0% (*n* = 8) of patients with a smaller body size. Post‐implantation programming predominantly consisted of a two‐zone setting, with conditional and shock zone thresholds of 200 and 240 bpm, respectively.

**TABLE 3:** Procedure details and device setting data.

	*n* = 96
Procedure details	
General anesthesia, *n* (%)	71/90 (78.9)
Implantation side, left, *n* (%)	83/89 (93.3)
Two incisions, *n* (%)	53/90 (58.9)
Tunneler bending, *n* (%)	8/89 (9.0)
Procedure time, min (range)	95 (78–129)
Device setting	
Sensing Vector, primary, *n* (%)	33/86 (38.4)
Sensing Vector, secondary, *n* (%)	32/86 (37.2)
Sensing Vector, alternate, *n* (%)	21/86 (24.4)
Shock Vector, standard, *n* (%)	86/86 (100)
Zone setting, two zone, *n* (%)	87/90 (96.7)
Conditional zone, bpm (range)	200 (200–200)
Shock zone, bpm (range)	240 (220–250)
Smart pass filter, on, *n* (%)	79/88 (89.8)

### 3.4 Follow‐Up Data

**FIGURE 2:**
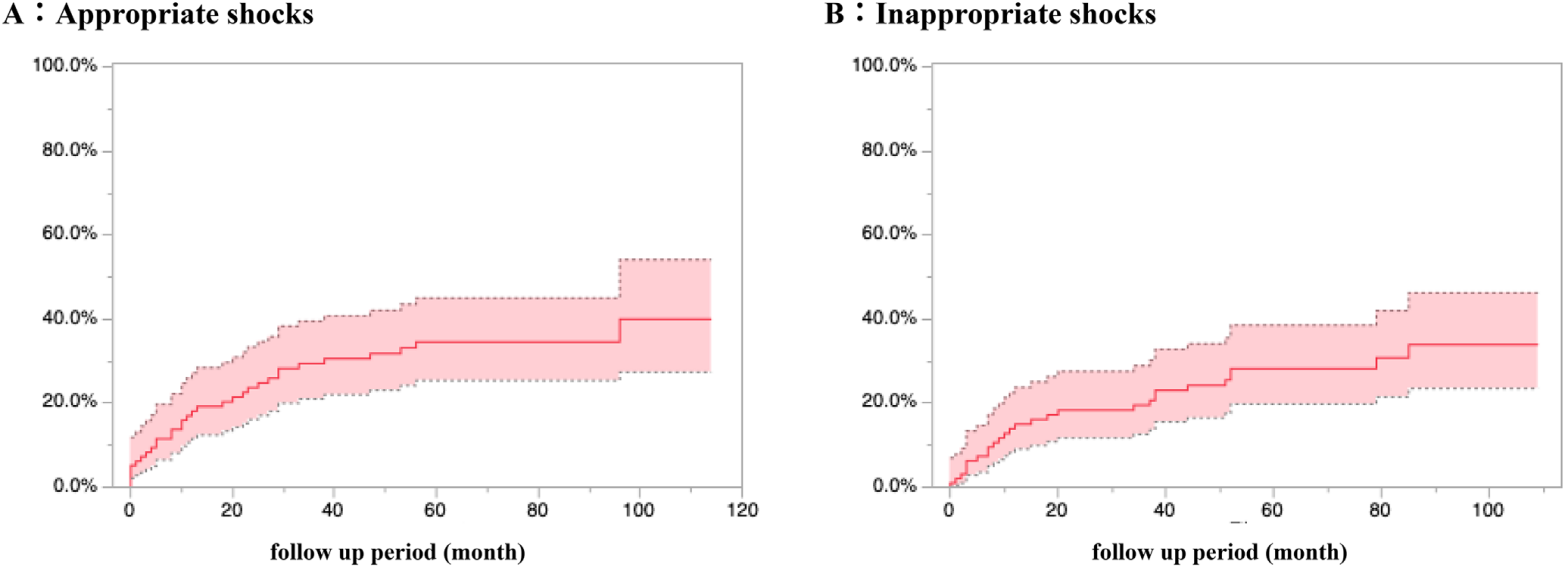
(A) depicts the Kaplan–Meier curve for appropriate shocks, with a total of 32 patients experiencing appropriate device therapy. (B) illustrates the Kaplan–Meier curve for inappropriate shocks, which occurred in 27 patients.

During a median follow‐up period of 70 months (IQR, 29.0–75.0 months), increases in height and body weight were observed; however, no significant changes in sensing vector suitability were identified (Table S1). Figure 2 shows the Kaplan–Meier curves for appropriate and inappropriate shocks. During follow‐up, appropriate shocks occurred in 32 patients (33.7%), whereas inappropriate therapy was observed in 27 patients (28.4%).

**FIGURE 3:**
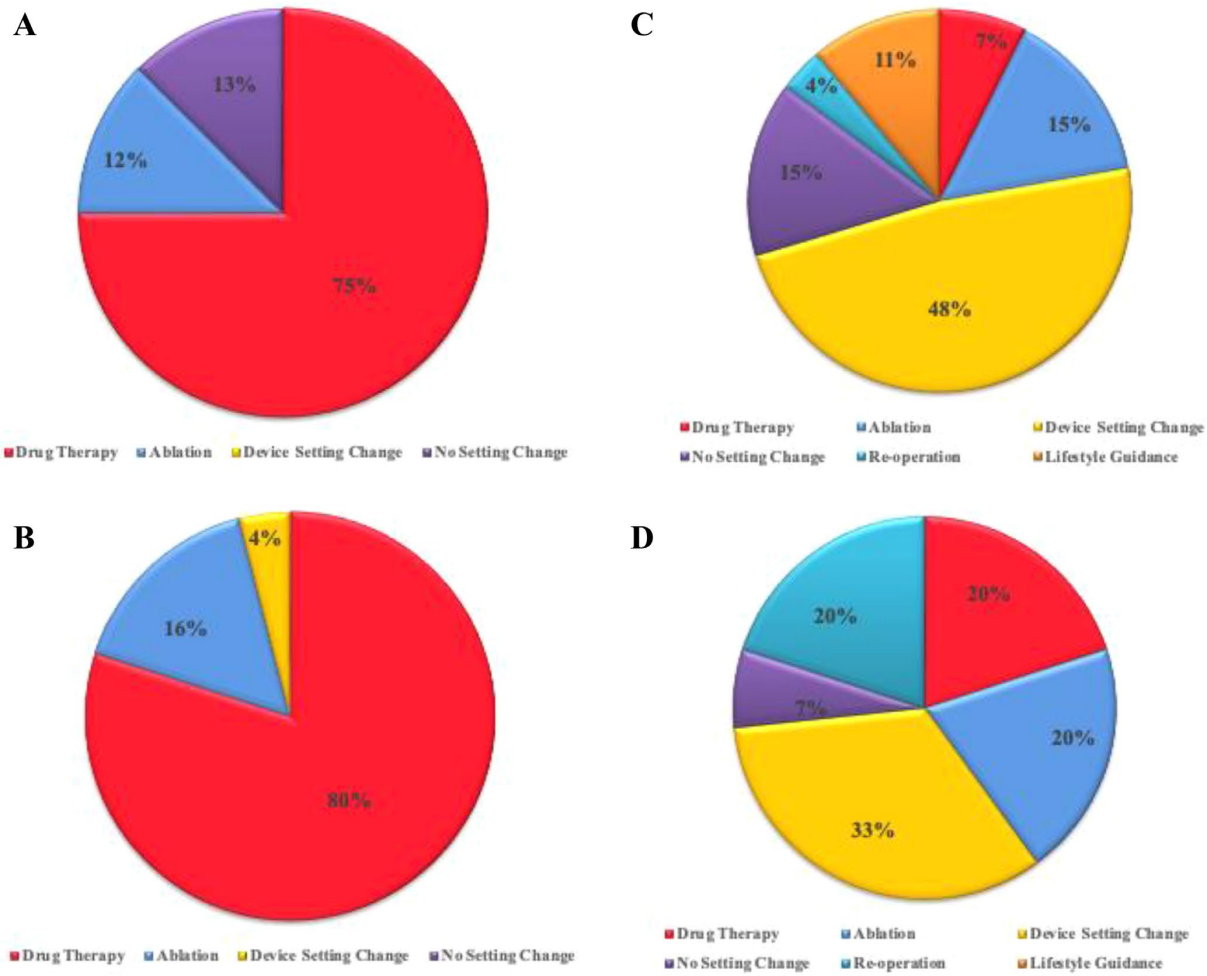
A (first shock) and B (subsequent shock) show the management strategies after appropriate therapy, in which drug therapy was the most frequently implemented intervention. C (first shock) and D (subsequent shock) show the management strategies after inappropriate therapy, where device setting modification was the most frequently performed intervention.

A total of 57 appropriate therapies (32 first shocks and 25 subsequent shocks) and 42 inappropriate therapies (27 first inappropriate shocks and 15 subsequent inappropriate shocks) were analyzed. Regarding management after appropriate shocks, drug therapy was the most frequently selected strategy after both the first (Drug therapy, *n* = 24 [75.0%]; Ablation, *n* = 4 [12.5%]; No setting change, *n* = 4 [12.5%]) and subsequent shocks (Drug therapy, *n* = 20 [80.0%]; Ablation, *n* = 4 [16.0%]; Device setting change, *n* = 1[4.0%]). In contrast, for inappropriate shocks, device setting change was the most commonly implemented intervention after both the first (Drug therapy, *n* = 2 [7.4%]; Ablation, *n* = 4 [14.8%]; Device setting change, *n* = 13 [48.1%], No setting change, *n* = 4 [14.8%]; Re‐operation, *n* = 1 [3.7%]; Lifestyle Guidance, *n* = 3 [11.1%]) and subsequent shocks (Drug therapy, *n* = 3 [18.8%]; Ablation, *n* = 3 [18.8%]; Device setting change, *n* = 5 [31.3%], No setting change, *n* = 1 [6.3%]; Re‐operation, *n* = 3 [18.8%]) (Figure 3).

**FIGURE 4:**
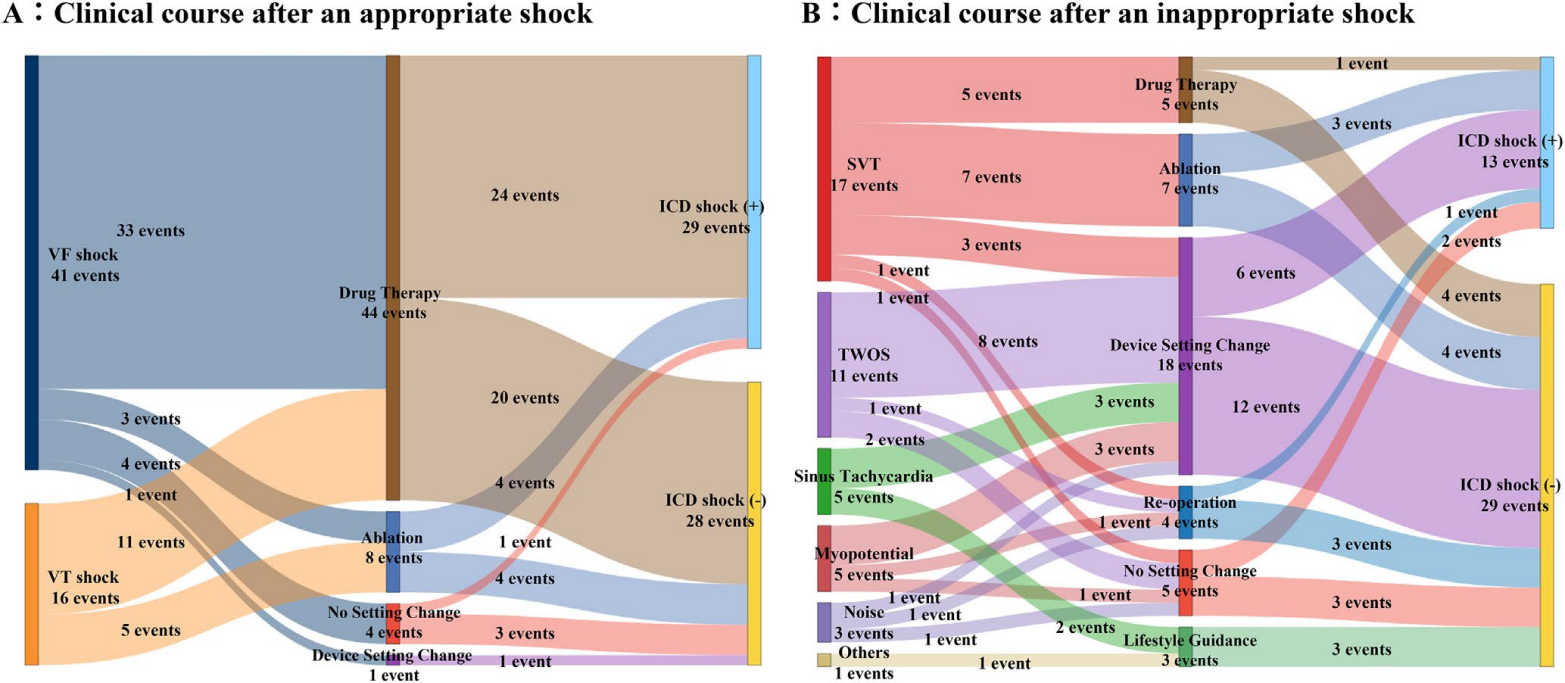
Sankey diagram summarizing clinical management strategies following 57 appropriate therapy events in 32 patients and 42 inappropriate therapy events in 27 patients, along with subsequent recurrence of device shocks. TWOS, T‐wave oversensing.

Figure 4 presents a Sankey diagram illustrating clinical management strategies following 57 appropriate shock events in 32 patients and 42 inappropriate shock events in 27, as well as the subsequent recurrence of device shocks. After appropriate shocks for VT/VF, intensification of drug therapy was the most frequently adopted management strategy, followed by catheter ablation. Intensified drug therapy successfully prevented recurrent device therapy in 45.5% of cases (20/44), while catheter ablation prevented recurrence in 50.0% of cases (4/8). Regarding inappropriate shocks, supraventricular tachycardia (SVT) was the most common underlying cause, followed by T‐wave oversensing (TWOS). One case of inappropriate shock was triggered by bathing in an electrically charged bath (classified as others). After inappropriate shocks, device setting change was the most frequently implemented intervention, and recurrent device therapy was avoided in 66.7% of cases following device setting change (12/18). Lifestyle guidance also prevented recurrent inappropriate therapy (3/3). These two non‐invasive interventions prevented recurrence in 71.4% of patients (15/21).

At the final follow‐up, pharmacological therapy had not been initiated in 23 patients (24.0%). Only one patient died during the follow‐up period. This patient was a 3‐year‐old boy with multiple congenital anomaly syndrome who underwent S‐ICD implantation for VF associated with LQTS. One year after implantation, he died from a non‐cardiac cause related to his underlying condition.

### 3.5 Device Related Events

Table 4 summarizes device‐related events. During the acute phase, one patient (1.0%) developed an infection that required device extraction. One case of inappropriate therapy was attributed to subcutaneous air entrapment, and lead repositioning was performed in one patient.

**TABLE 4:** Device related events.

Acute phase events	
Acute device infection, *n* (%)	1/96 (1.0)
Air contamination, *n* (%)	1/96 (1.0)
Lead repositioning, *n* (%)	1/96 (1.0)
Chronic phase events	
Inappropriate shock, *n* (%)	27/95 (28.4)
Recalled device, *n* (%)	11/91 (12.1)
Early battery depletion, *n* (%)	4/95 (4.2)
Lead repositioning, *n* (%)	3/95 (3.2)
Chronic device infection, *n* (%)	1/95 (1.1)
Device upgrade, *n* (%)	2/95 (2.1)

During the chronic phase, 11 patients (12.1%) were identified as having recalled devices, and premature battery depletion was observed in 4 patients (4.2%). Lead repositioning due to growth‐related lead displacement was required in three patients. On the other hand, device‐related infection was observed in only one patient (1.1%). Upgrade to a TV‐ICD was required in one patient with sarcoidosis and in one patient with CPVT. There were no cases of lead fracture during follow‐up.

## 4 Discussion

### 4.1 Major Findings

The major findings of the present study are as follows:

1. To our knowledge, this represents one of the longest follow‐up analyses of S‐ICD therapy in pediatric patients reported to date.
2. The majority of ICD implantations were performed for secondary prevention of VF, with primary prevention accounting for only 11% of cases.
3. Sensing vector suitability did not show any significant changes over a 70‐month follow‐up period.
4. An appropriate shock rate of 33.7% demonstrated the clinical effectiveness of S‐ICD therapy for SCD prevention, although the incidence of inappropriate therapy remained 28.4%.
5. Device‐related infection was rare, with only one case in the acute phase and one in the chronic phase, and no cases of bacteremia were observed.
6. Despite therapeutic interventions following appropriate therapy, recurrent device therapy occurred in 50.9% of cases. In contrast, after inappropriate therapy, therapeutic interventions successfully prevented recurrence in 69.0% of patients.

### 4.2 Baseline Characteristics at Implantation

In the present study, the majority of ICD implantations were performed for secondary prevention of VF, whereas primary prevention accounted for 11% of cases. The proportions of primary and secondary prevention were comparable to those reported in the previous study [21]. In addition, no patients aged 12 years or younger underwent ICD implantation for primary prevention, which is also consistent with the prior report. Regarding underlying conditions, secondary prevention for idiopathic VF was the most common indication, followed by HCM. These findings highlight the importance of SCD in patients with HCM.

Recent studies have reported the widespread efficacy of S‐ICD therapy across a broad spectrum of underlying pathologies, ranging from channelopathies to cardiomyopathies [22, 23]; however, these findings have been predominantly limited to adult populations. Our results extend these findings to younger populations, suggesting that S‐ICD therapy is highly effective and safe for pediatric patients with both channelopathies and cardiomyopathies.

Furthermore, our findings show the safety of S‐ICD in younger patients, suggesting that primary prevention may be considered in patients with HCM aged 13 years or older using S‐ICD.

### 4.3 Sensing Vectors Suitability During the Mid‐Term Follow‐Up

In the SAVE‐CHILDREN study, no changes in sensing vector suitability were observed over a median follow‐up period of 27 months [17]. In the present study, despite a substantially longer median follow‐up of 70 months, sensing vector suitability similarly remained unchanged. Because the median age at implantation in our cohort was approximately 14.5 years—corresponding to the pubertal period—if no changes in sensing vectors were observed during the early post‐implantation phase, subsequent effects of somatic growth on vector suitability were likely minimal. Accordingly, the relatively limited changes in body size after implantation may explain the sustained stability of sensing vector suitability over long‐term follow‐up.

### 4.4 Midterm Efficacy and Safety of S‐ICD for Patients Less Than 18 Years Old

In the PRAETORIAN trial, which reported outcomes in an adult population, the incidence of appropriate device therapy was 15.1% [4], whereas our study revealed that an appropriate shock was observed in the 32 patients (33.7%). This difference may be explained by the fact that secondary prevention accounted for only 18.8% of patients in the PRAETORIAN trial, whereas in the present study, 80.2% of patients underwent S‐ICD implantation for secondary prevention. The substantially higher proportion of secondary‐prevention cases in our cohort likely contributed to the higher rate of appropriate therapy observed.

Although pharmacological therapy was administered in 76% of patients, a high incidence of appropriate device therapy was still observed. These findings suggest that device‐based secondary prevention may play a particularly important role in pediatric patients who have experienced SCD or life‐threatening ventricular arrhythmias.

The battery longevity of ICDs is shorter than that of pacemakers [24], and long‐term device use often necessitates multiple generator replacements, raising concerns regarding device‐related infection [10]. In the present study, however, only one case of device infection occurred during the chronic phase, indicating a relatively low infection rate even during mid‐term follow‐up. Previous studies have reported that generator implantation in the intermuscular space reduces device‐related complications compared to implantation in the subcutaneous space [25]. Because pediatric patients typically have thin subcutaneous tissue, S‐ICD generators are frequently implanted in the intermuscular space in this population, which likely contributed to the low incidence of device‐related complications observed in our study.

In addition, although recent studies have reported improved durability of ICD leads [26, 27], pediatric patients are highly active, and previous reports have demonstrated a higher incidence of lead fracture associated with transvenous leads in the pediatric population [11]. In contrast, no cases of subcutaneous lead fracture were observed in the present study, consistent with findings in adult populations [28], supporting the durability and safety of subcutaneous leads in pediatric patients. However, lead repositioning was required in three patients due to growth‐related lead displacement, underscoring the importance of careful monitoring of lead position during long‐term device follow‐up in pediatric populations.

### 4.5 Management and Outcomes After Appropriate and Inappropriate Shocks

Both appropriate and inappropriate ICD shocks have been reported to be associated with patients' mortality and re‐hospitalization [29]. In addition, patients who receive shocks experience a decline in quality of life (QOL) in many aspects, including physical and emotional functioning [30]. Based on these findings, device programming and pharmacologic therapy aimed at shock reduction are considered important components of patient management.

In the present study, which included a high proportion of secondary‐prevention cases, recurrent device therapy was observed in a substantial proportion of patients (50.9%) despite therapeutic interventions following appropriate shock. Intensified drug therapy successfully prevented recurrent device therapy in 45.5% of cases, while catheter ablation prevented recurrence in 50.0% of cases. Given the procedural risks associated with catheter ablation, these findings suggest the possibility that optimization of pharmacological therapy may be a reasonable initial strategy following appropriate device therapy.

The most common cause of inappropriate therapy was SVT, followed by TWOS. Since previous studies have reported that the Smart Pass filter is useful in reducing TWOS [31, 32], and it was implemented in 89.8% of patients in this study, this likely explains why SVT became the leading cause of inappropriate shocks. In patients who underwent device setting change, recurrent inappropriate therapy was successfully prevented in 66.7% of cases, highlighting the importance of appropriate device optimization. In addition, inappropriate therapies triggered by sinus tachycardia or bathing in an electrically charged bath were successfully prevented through lifestyle guidance alone. These findings underscore the importance of providing appropriate ICD‐related education to pediatric patients to reduce the risk of inappropriate therapy.

### 4.6 Device Selection for the Prevention in the Younger Patients

Several device‐based therapeutic options are available for the prevention of SCD in pediatric patients: TV‐ICDs, S‐ICDs, EV‐ICDs, and non‐transvenous ICD systems with epicardial leads. Although current guidelines provide recommendations regarding indications for ICD implantation, they do not specify criteria for device selection [33]. Selection among these devices should be individualized, taking into account device suitability, the need for pacing therapy, and the risk of long‐term complications. In patients with congenital heart disease who have residual intracardiac shunts after surgical repair, the use of TV leads may be contraindicated because of concerns regarding thromboembolic complications, necessitating consideration of S‐ICD or EV‐ICD systems. Although sensing vector suitability is a critical issue when selecting an S‐ICD, the present study demonstrated that only a small minority of patients failed pre‐screening in all vectors, while the majority were eligible for S‐ICD therapy. These findings indicate that S‐ICD represents an important therapeutic option for pediatric patients in whom intravascular lead placement is undesirable or not feasible. Furthermore, no patients in the present study required upgrade to a TV‐ICD because of bradycardia during follow‐up. Although selection bias cannot be excluded—given that patients with baseline bradycardia were less likely to receive an S‐ICD—our findings suggest that, in pediatric patients without pacing requirements at implantation, S‐ICD therapy may be considered a first‐line option, even in those with inherited arrhythmia syndromes.

### 4.7 Study Limitations

This study has several limitations. First, this was a retrospective observational study, and we did not directly compare outcomes among different types of ICDs, such as TV‐ICDs or EV‐ICDs, within the study cohort. Therefore, prospective studies including multiple device types are warranted in the future to allow direct comparison of clinical outcomes. Second, previous studies have suggested that changes in body size associated with growth may affect the defibrillation threshold (DFT) [34]. However, in the present study, we were unable to evaluate changes in DFT at the time of device replacement. Consequently, the longitudinal changes in DFT in our cohort remain unknown.

## 5 Conclusions

S‐ICD therapy demonstrated favorable mid‐term efficacy and safety in pediatric patients, with a low incidence of device‐related infections. However, inappropriate shocks were not uncommon, underscoring the importance of appropriate post‐shock management to reduce recurrent device therapies.

## Author Contributions

H.M., N.S., T.N., K.N., H.F., and H.H., study conception and design; O.I., Y.I., S.A., J.N., S.W., S.O., H.S., H.F., J.K., T.C., T.H., A.O., H.K., T.S., T.O., S.S., K.Y., H.K., N.T., K.K., M.M., J.M., S.T., K.I., H.G., T.M., T.W., M.T., T.S., Y.I., S.S., T.N., M.T., T.M., T.I., S.S., M.F., M.K., R.H., S.F., H.S., Y.Y., I.M., K.K., E.N., Y.N., T.S., Y.A., K.N. (Nishizaki), K.N. (Nakamura), N.N., M.Y., Y.I., Y.O., Y.K., M.K. (Nakano), M.K. (Kito), Y.I., S.H., T.K., K.M., M.N., W.S., D.K., T.N., N.T., and K.M., contributed to the data collection, manuscript revision, and data analysis; T.K., Y.I., R.K., and N.S. provided the study supervision.

## Funding

The authors have nothing to report.

## Disclosure

Hitoshi Mori received lecture fees from Biosense Webster Japan and Boston Scientific Japan. Our department received grant support from Boston Scientific Japan and Abbott Medical Japan.

## Conflicts of Interest

The authors declare no conflicts of interest.

## Supporting information


**Figure S1:** Age distribution according to indications for ICD implantation (primary prevention, secondary prevention for VT, and secondary prevention for VF). Primary prevention ICD implantation was performed only in patients aged 13 years or older, whereas all ICD implantations in patients aged 12 years or younger were indicated for secondary prevention.

**Table S1:** Device follow up data.

## Data Availability

The data underlying this article cannot be shared publicly due to our IRB policy. However, the data will be shared on reasonable request to the corresponding author.
