# Supplementary figures and images for: Midterm Efficacy of SubcutAneous Implantable CardioVErter‐Defibrillator in ≤ 18 Year‐Old CHILDREN (SAVE CHILDREN‐II Registry)
Source: J Arrhythm. 2026 Jul 13;42(4):e70420. doi: 10.1002/joa3.70420 (PMC13365360; doi:10.1002/joa3.70420)

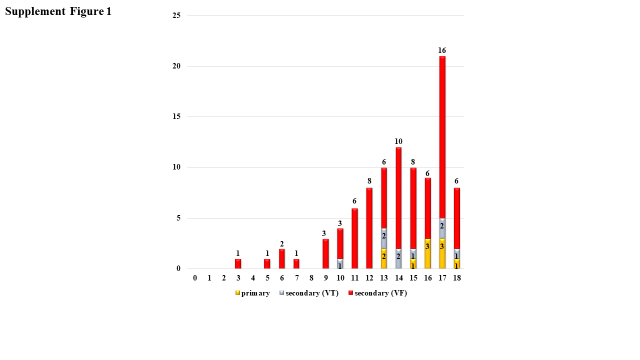

Supplement: Supplementary file 1 — Figure S1: Age distribution according to indications for ICD implantation (primary prevention, secondary prevention for VT, and secondary prevention for VF). Primary prevention ICD implantation was performed only in patients aged 13 years or older, whereas all ICD implantations in patients aged 12 years or younger were indicated for secondary prevention. [file JOA3-42-e70420-s001.docx]
