# Supplementary material for: Midterm Efficacy of SubcutAneous Implantable CardioVErter‐Defibrillator in ≤ 18 Year‐Old CHILDREN (SAVE CHILDREN‐II Registry)
Source: J Arrhythm. 2026 Jul 13;42(4):e70420. doi: 10.1002/joa3.70420 (PMC13365360; doi:10.1002/joa3.70420)
Supplement: Supplementary file 2 — Table S1: Device follow up data. [file JOA3-42-e70420-s002.docx]

**Supplement Table 1. Device follow up data**

|  | Initial follow up | Last follow up | p |
| --- | --- | --- | --- |
| Patient age, years | 14.5 (12.0-17.0) | 20.0 (17.0-23.0) | <0.0001 |
| Body height, cm | 160.3 (149.6-170.0) | 164.9 (159.2-173.4) | 0.0016 |
| Body weight, kg | 50.0 (43.0-57.7) | 55.1 (48.7-65.0) | 0.0001 |
| Primary (spine position), pass, n (%) | 50/55 (90.9) | 36/39 (92.3) | 0.81 |
| Secondary (spine position), pass, n (%) | 50/51 (98.0) | 33/34 (97.1) | 0.77 |
| Alternate (spine position), pass, n (%) | 47/52 (90.4) | 33/35 (94.3) | 0.51 |
| Primary (sitting position), pass, n (%) | 41/48 (85.4) | 27/30 (90.0) | 0.56 |
| Secondary (sitting position), pass, n (%) | 42/44 (95.5) | 25/25 (100) | 0.28 |
| Alternate (sitting position), pass, n (%) | 39/46 (84.8) | 24/26 (92.3) | 0.35 |
